# Supplementary material for: A comparison of the attractiveness of flowering plant blossoms versus attractive targeted sugar baits (ATSBs) in western Kenya
Source: PLoS One. 2023 Jun 6;18(6):e0286679. doi: 10.1371/journal.pone.0286679 (PMC10243617; doi:10.1371/journal.pone.0286679)
Supplement: S4 File — (DOCX) [file pone.0286679.s004.docx]

**TESTING MOSQUITO ATTRACTION TO FLOWERS & ATSBs IN A SEMI-FIELD STRUCTURE**

**OBJECTIVE**

To determine the attraction of malaria vectors to different flowering plants in a semi-field area in order to estimate potential competition for ATSB.

**DEFINITIONS**

ATSB: Attractive Targeted Sugar bait

SFs: Semi-field structures

SOP: Standard Operating procedure

**MATERIALS**

1. Plastic jar

2. Plastic nets 70 by 70 cm with a mesh size of 0.8cmx0.2cm

3. Glue (Tangle Foot, Tel Aviv Israel)

4. 20cm Wooden sticks

5. Regular Water

6. 20L plastic bucket

7. Hard gloves

8. Panga/Matchet

9. Field collected *An. arabiensis*, *An. gambiae*, and *An. funestus* mosquitoes.

10. Forceps

11. 1.5 Eppendorf tubes

12.70% ethanol/Desiccator (silica gel)

13. Plastic seals

14. Fresh flowers to be evaluated for attractancy

15. ATSB versions

**EXPERIMENTAL DESIGN**

**Entry**

**Key**

**Semi-field design**

**-**Represents positions of test flowers & ATSB and the controls in the screen house

-Mosquito release point in the screen house

**Arrows** ( ) represent the direction of rotation of test materials

This experiment will be conducted in three semi-field structures (SFs). 16 plants will be tested in two sets for attraction in the semi-field structures. Each semi-field structure will receive three freshly picked flowers of different plant species and ad-libitum water as control. Each plant is to be rotated within the SFs in the 4 indicated positions. Newly emerged F1 generation of wild *Anopheles arabiensis*, *An. gambiae* and *An. funestus* mosquito pre-starved 24 hours will be released at a central point (see diagram above). The six most attractive plants will be compared with the ATSB products for potential competition.

**PROCEDURE**

1. Set up the experiment in each of the four corners of the SFs, maintaining a distance of 10m by 8 meters between the test materials.
2. At the semi-field structure, corners marked A, B, C & D, bury in the ground an empty 1.5L plastic jar and fill it with water from tap water held in the 20-liter bucket.

3. Firm up the soil around the plastic jar to consolidate it.

4. Cut and roll the 70cm x 70cm plastic net into a cylinder and fix it with plastic seals to form a trap. Close the top part of the cylinder with the same material.

5. Place the cylinder net around the plastic jar to enclose it, fixing it well with two 20cm long wooden sticks to the ground to prevent toppling in stormy weather

5. Place four traps per semi-field structure at a time

6. Place ad-libitum water control in one corner of the 3 Semi-field structures

7. Collect fresh flowers from flowering plants from the study area, ensuring to put on protective industrial gloves. Gently cut the identified plants and flowers with a panga.

8. Randomize the flowering plants for each of the 3 semi-field structures.

9. In each of the cylinder nets, place a bunch of freshly cut flowers approximately 50 cm in length or ATSB version to be tested

10. Apply tangle foot glue on the outer surface of plastic cylinder nets (Tel Aviv Israel)

10. Release 600 (300 males and 300 females), 3-5 days post-emergence of field-collected *Anopheles arabiensis, An. gambiae* or 200 of F1 *An. funestus* mosquitoes at a central release point within the SFs. Release the samples in the evening between 1700-1800 hours and leave the experiment overnight.

11. On a consecutive morning gently collect all trapped mosquitoes from the glued-netted traps with a pair of forceps and place them in the Eppendorf tubes

12. Count, sex, and record the data in a data form. Preserve them in Eppendorf tubes containing 70% ethanol or desiccated

13. Use at least four replicates for each flower/test materials

14. Identify each plant at the species level.

**REFERENCES**

1. Müller, G.C.; Beier, J.C.; Traore, S.F.; Toure, M.B.; Traore, M.M.; Bah, S.; Doumbia, S.; Schlein, Y. Field experiments of *Anopheles gambiae* attraction to local fruits/seedpods and flowering plants in Mali to optimize strategies for malaria vector control in Africa using attractive toxic sugar bait methods. *Malar. J.* **2010**, *9*, 262.
