# Supplementary material for: A comparison of the attractiveness of flowering plant blossoms versus attractive targeted sugar baits (ATSBs) in western Kenya
Source: PLoS One. 2023 Jun 6;18(6):e0286679. doi: 10.1371/journal.pone.0286679 (PMC10243617; doi:10.1371/journal.pone.0286679)
Supplement: S5 File — (DOCX) [file pone.0286679.s005.docx]

**SEMI-FIELD STRUCTURE & THE 4 TRAP LOCATIONS**


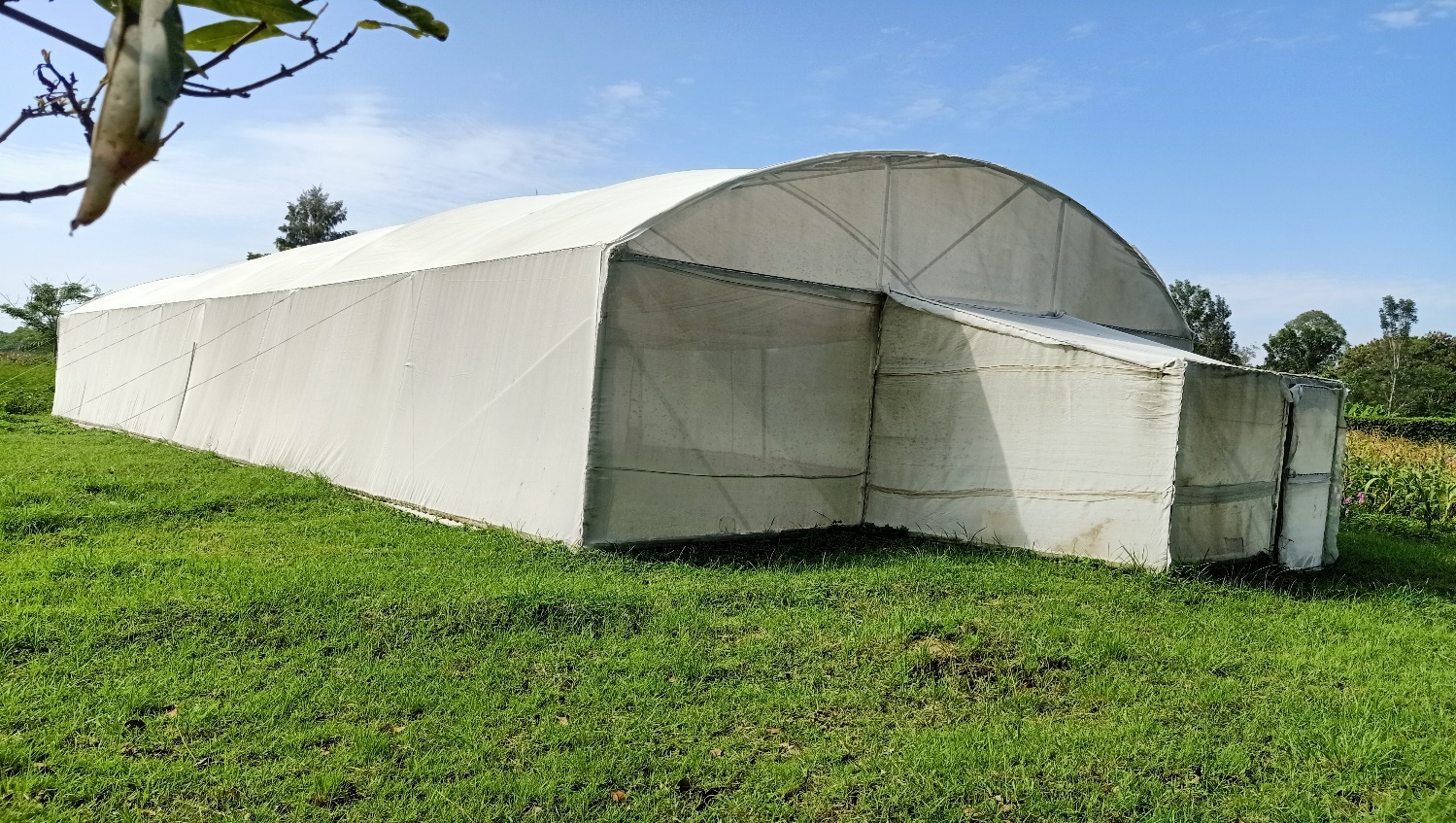


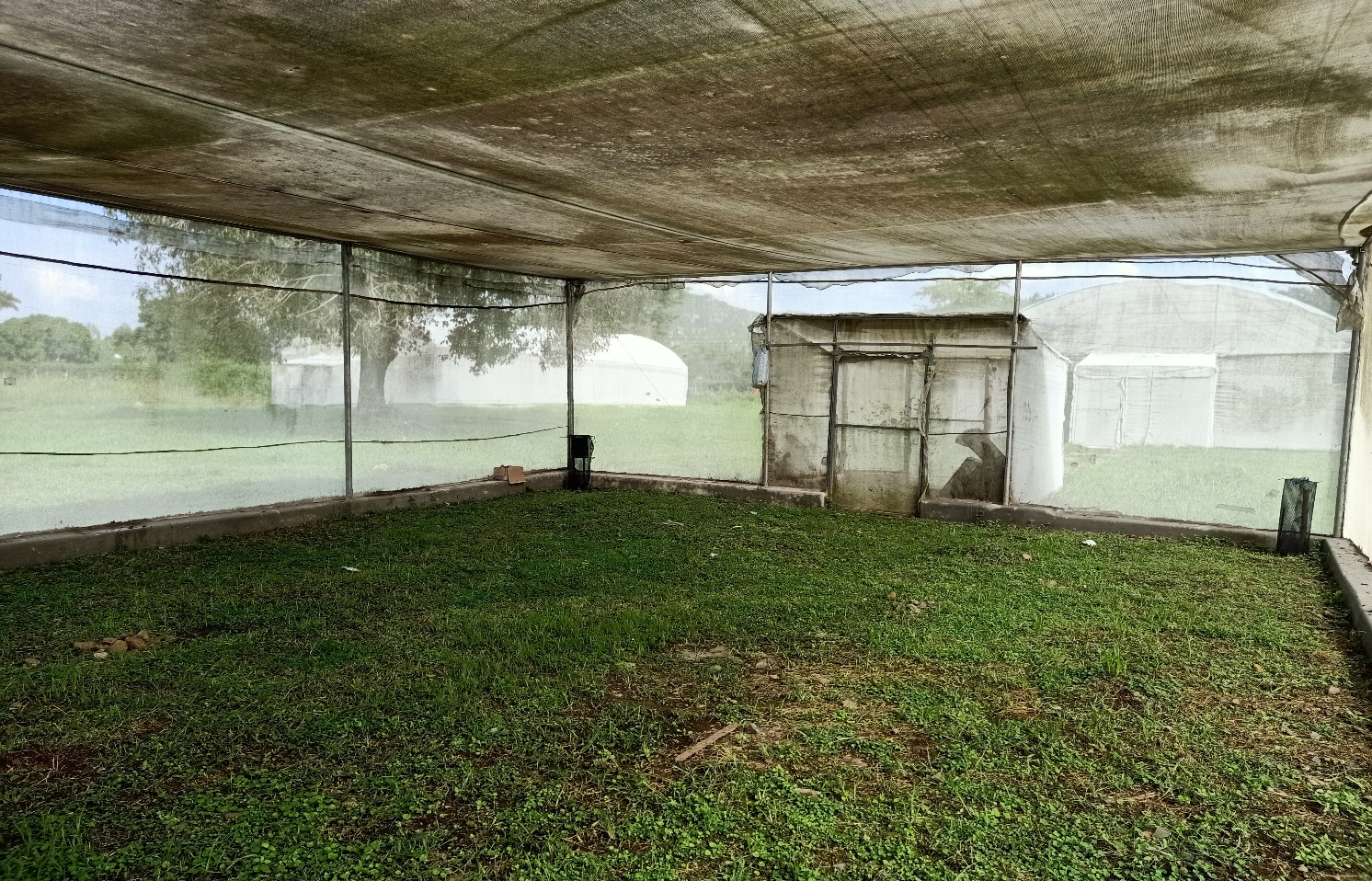


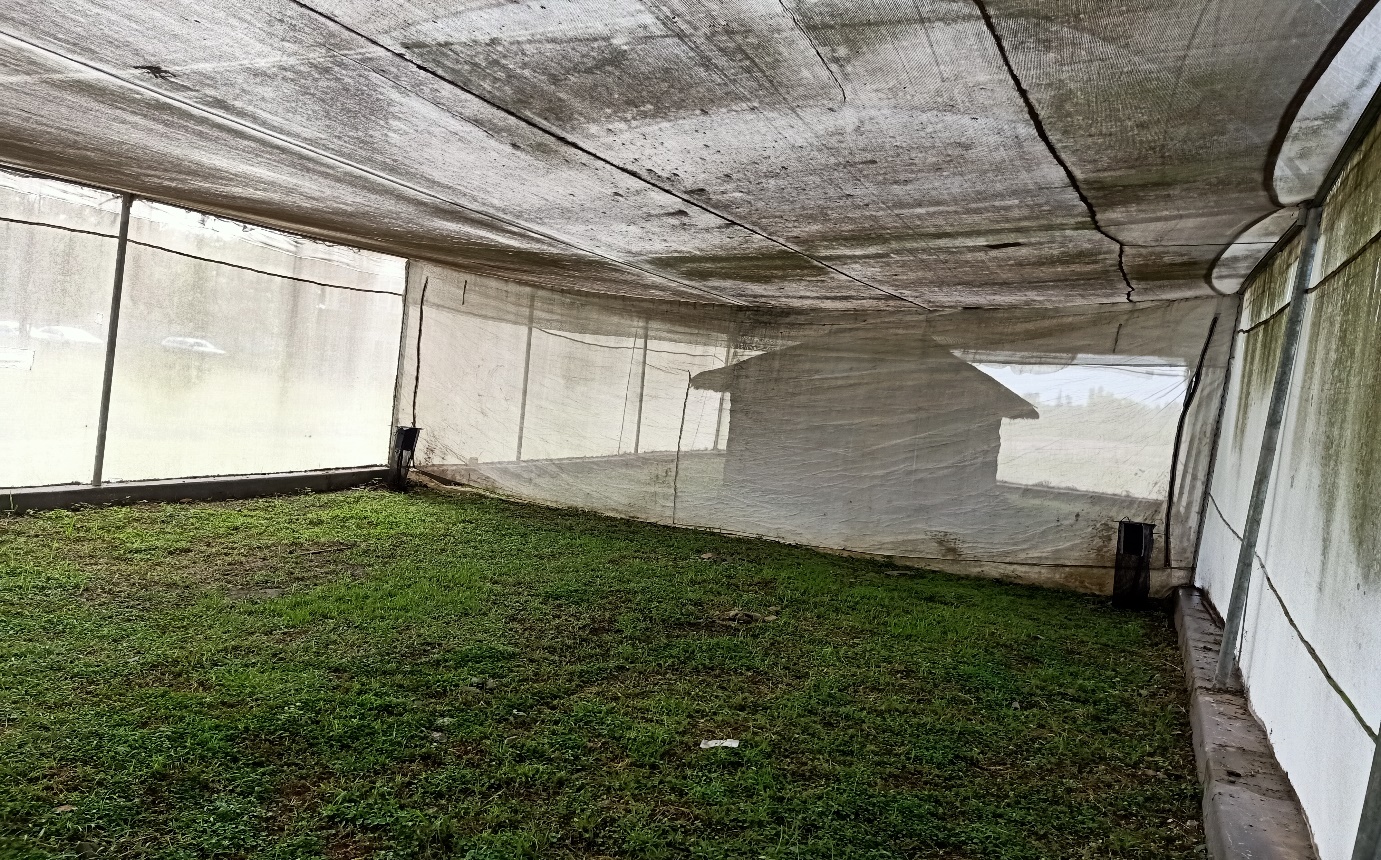


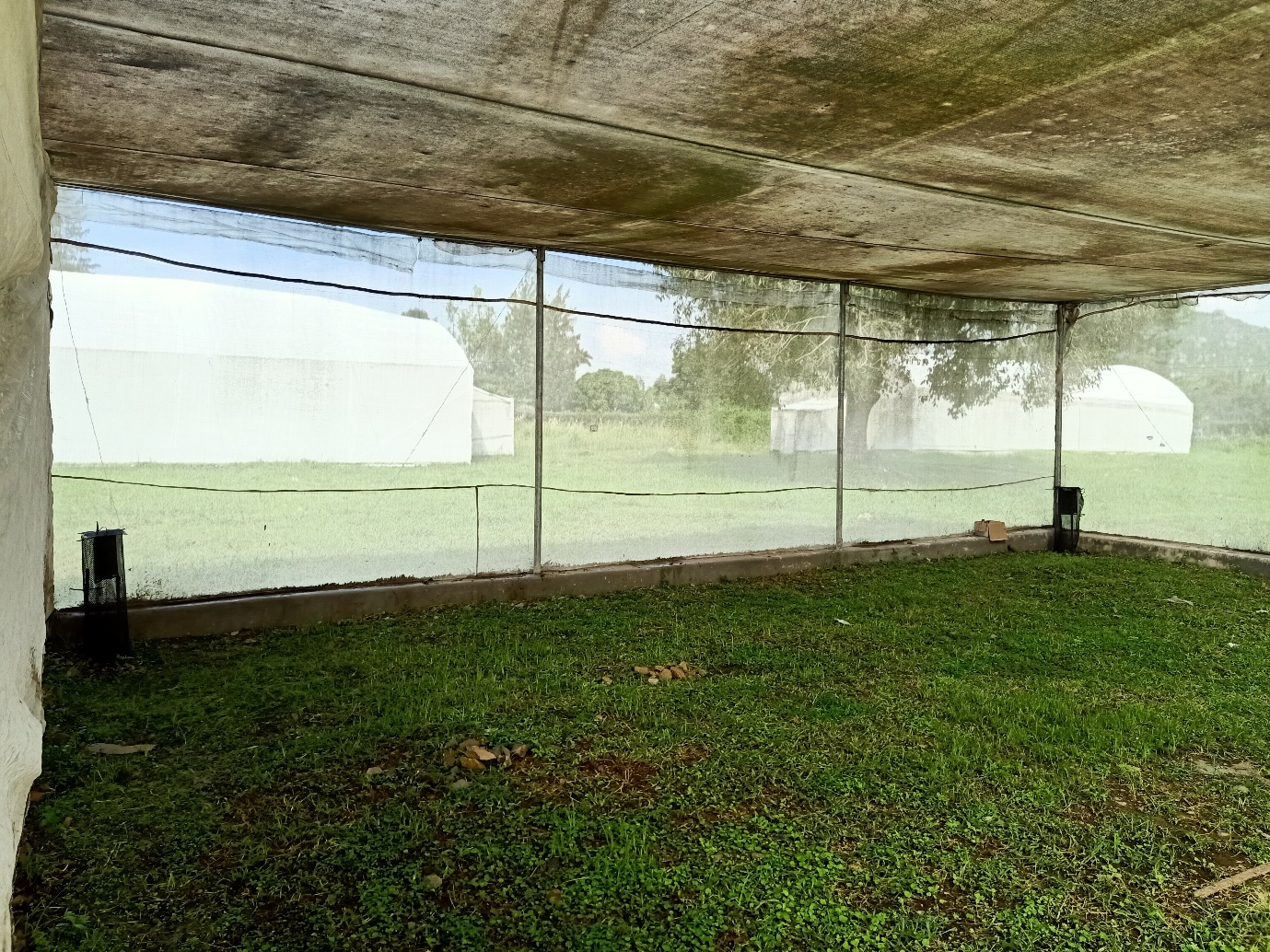


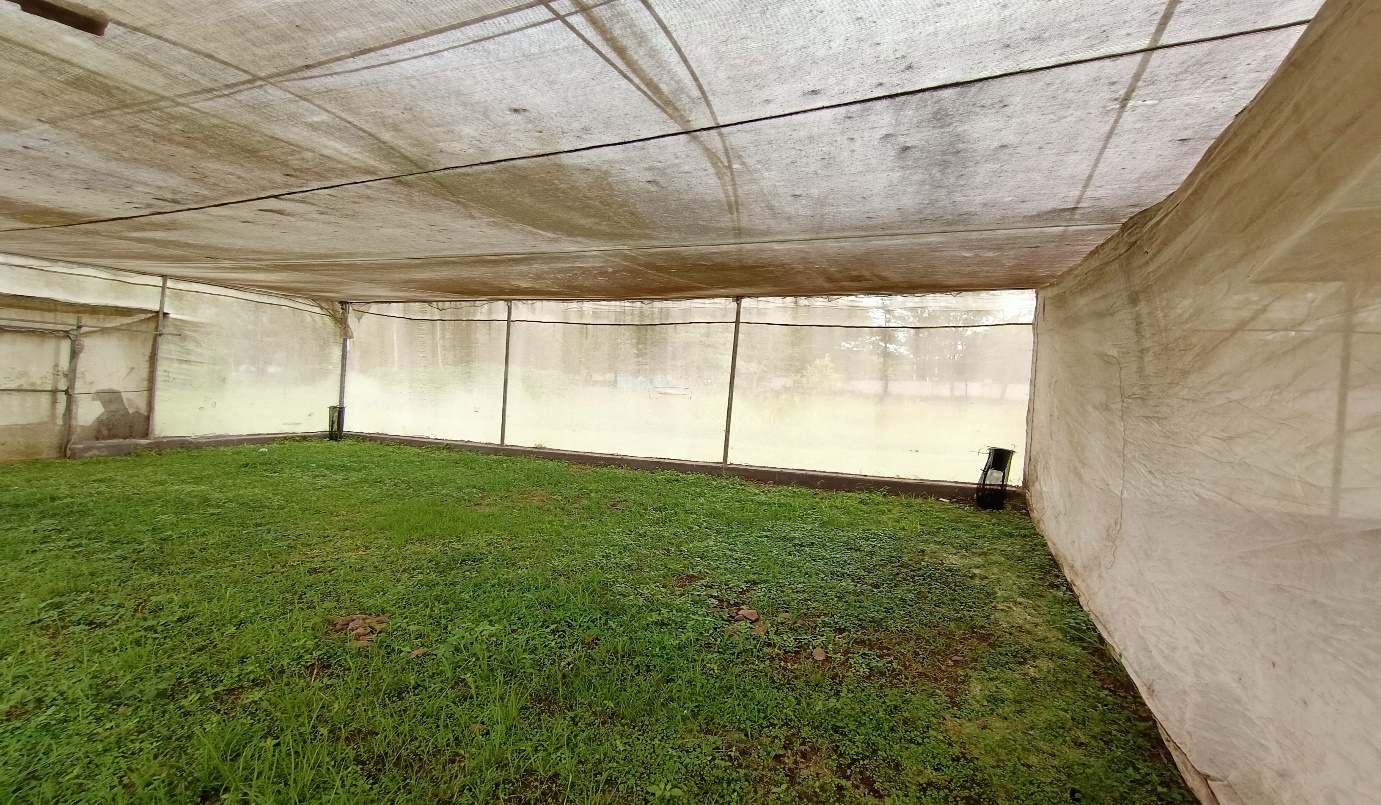


-The semi-field area measured 10 x 8 metres (Length x width)

- The first picture is the outer look of one of the three similar semi-field structures used in the experiment. The subsequent pictures represent the four sides indicating where the glue-netted traps are located during tests.

-The mosquito release area is at the central point of the semi-field section.
