# Supplementary material for: A comparison of the attractiveness of flowering plant blossoms versus attractive targeted sugar baits (ATSBs) in western Kenya
Source: PLoS One. 2023 Jun 6;18(6):e0286679. doi: 10.1371/journal.pone.0286679 (PMC10243617; doi:10.1371/journal.pone.0286679)
Supplement: Attachment — Submitted filename: Response to Reviewers 27April2023.docx [file pone.0286679.s007.docx]

**Response to Reviewers.**

Journal Requirements:

Please review your reference list to ensure that it is complete and correct. If you have cited papers that have been retracted, please include the rationale for doing so in the manuscript text, or remove these references and replace them with relevant current references. Any changes to the reference list should be mentioned in the rebuttal letter that accompanies your revised manuscript. If you need to cite a retracted article, indicate the article’s retracted status in the References list and also include a citation and full reference for the retraction notice.

*Response: The reference list is complete and correct based on my review. No retracted paper was cited. Therefore, no changes were made to the list.*

Reviewer #2: Major comments
Please describe all versions of ATSBS under subheading in “Materials and Methods” section.

*Response-We’ve documented the notable differences and similarities in the three versions of the ATSB tested. Again, via personal information from the manufacturer, we learnt that despite the similarity in bait formulation, there were a few differences noted in ( v1.1 & 1.1.2) that ultimately led to the manufacture of the final version 1.2 used in the main trial. These differences are in the membrane texture, bait retention ability, and longevity but this is information we are only reporting based on what is disclosed to us by the manufacturer*

Figure 5 showed significant result against An. arabiensis in all test groups, however, results section showed non-significant p-values in Lane 251 and 253. Please check and correct the figure.

*Response-The error in figure five was fixed. See attached figure 5.*
Lane no 266: Mentioned “non-significant relationships not shown” however results showed (lane 253 and 255) non-significant ‘p’ values.

*Response-This was corrected after fixing figure 5*

Minor comments
Remove the underline symbol below degree Celsius (27-30 ºC)

*Response – this was corrected*

I find different terminology used in MS for flowering plants. I suggest single terminology would be better.

*Response - The flowering part of a plant that will form the seeds or fruit is called a blossom and synonyms include bloom and flower. It really doesn’t alter the meaning of a flower.*

Correct the typo error in “An. Arabiensis”

*Response: The typo was corrected*

Lane 271: Current the typo error “ATSB 1.2 Mangifera indica”

*Response - In experiment 3 the ATSB version tested was labeled v1.1.2 which differs in labeling to the final ATSB version 1.2. The two versions are labeled so in the MS.*

Please ensure that you refer to Figure 4 in your text as, if accepted, production will need this reference to link the reader to the figure.

*Response: We are sorry for this error of omission; we include the reference along with that for Fig 3. Line 256*
